# Structure-Based Design and in Silico Screening of Virtual Combinatorial Library of Benzamides Inhibiting 2-trans Enoyl-Acyl Carrier Protein Reductase of *Mycobacterium tuberculosis* with Favorable Predicted Pharmacokinetic Profiles

**DOI:** 10.3390/ijms20194730

**Published:** 2019-09-24

**Authors:** Koffi Charles Kouman, Melalie Keita, Raymond Kre N’Guessan, Luc Calvin Owono Owono, Eugene Megnassan, Vladimir Frecer, Stanislav Miertus

**Affiliations:** 1 Laboratoire de Physique Fondamentale et Appliquée (LPFA), University of Abobo Adjamé (now Nangui Abrogoua), Abidjan 02, Côte d’Ivoire; koumank8@gmail.com (K.C.K.); keitamelalie@yahoo.fr (M.K.);; 2 International Centre for Theoretical Physics, ICTP-UNESCO, Strada Costiera 11, I-34151 Trieste, Italy; lcowono@yahoo.fr; 3 Department of Physics, Ecole Normale Supérieure, University of Yaoundé I, P.O. Box 47, Yaoundé 1, Cameroon; 4 International Centre for Applied Research and Sustainable Technology, SK-84104 Bratislava, Slovakia; vladimir.frecer@gmail.com (V.F.); stanislav.miertus@icarst.org (S.M.); 5 Laboratoire de Cristallographie—Physique Moléculaire, University of Cocody (now Felix Houphouët-Boigny), Abidjan 22, Côte d’Ivoire; 6 Laboratoire de Chimie Organique Structurale et Théorique, University of Cocody (now Felix Houphouët-Boigny), Abidjan 22, Côte d’Ivoire; 7 Department of Physical Chemistry of Drugs, Faculty of Pharmacy, Comenius University in Bratislava, SK-83232 Bratislava, Slovakia; 8 Department of Biotechnologies, Faculty of Natural Sciences, University of SS. Cyril and Methodius, SK-91701 Trnava, Slovakia

**Keywords:** Tuberculosis, N-benzyl-4-((heteroaryl)methyl)benzamides, 2-trans enoyl-acyl carrier protein reductase, molecular modeling, QSAR models, pharmacophore, combinatorial library, in silico screening, ADME properties prediction

## Abstract

Background: During the previous decade a new class of benzamide-based inhibitors of 2-trans enoyl-acyl carrier protein reductase (InhA) of Mycobacterium tuberculosis (Mt) with unusual binding mode have emerged. Here we report in silico design and evaluation of novel benzamide InhA-Mt inhibitors with favorable predicted pharmacokinetic profiles. Methods: By using in situ modifications of the crystal structure of N-benzyl-4-((heteroaryl)methyl) benzamide (BHMB)-InhA complex (PDB entry 4QXM), 3D models of InhA-BHMBx complexes were prepared for a training set of 19 BHMBs with experimentally determined inhibitory potencies (half-maximal inhibitory concentrations IC50exp). In the search for active conformation of the BHMB1-19, linear QSAR model was prepared, which correlated computed gas phase enthalpies of formation (∆∆HMM) of InhA-BHMBx complexes with the IC50exp. Further, taking into account the solvent effect and entropy changes upon ligand, binding resulted in a superior QSAR model correlating computed complexation Gibbs free energies (∆∆Gcom). The successive pharmacophore model (PH4) generated from the active conformations of BHMBs served as a virtual screening tool of novel analogs included in a virtual combinatorial library (VCL) of compounds containing benzamide scaffolds. The VCL filtered by Lipinski’s rule-of-five was screened by the PH4 model to identify new BHMB analogs. Results: Gas phase QSAR model: −log_10_(IC_50_^exp^) = *p*IC_50_^exp^ = −0.2465 × ∆∆H_MM_ + 7.95503, R^2^ = 0.94; superior aqueous phase QSAR model: *p*IC_50_^exp^ = −0.2370 × ∆∆G_com_ + 7.8783, R^2^ = 0.97 and PH4 pharmacophore model: $p{{\mathrm{IC}}_{50}}^{\exp}$ = 1.0013 × *p*${{\mathrm{IC}}_{50}}^{\exp}$ − 0.0085, R^2^ = 0.95. The VCL of more than 114 thousand BHMBs was filtered down to 73,565 analogs Lipinski’s rule. The five-point PH4 screening retained 90 new and potent BHMBs with predicted inhibitory potencies IC_50_^pre^ up to 65 times lower than that of BHMB1 (IC_50_^exp^ = 20 nM). Predicted pharmacokinetic profile of the new analogs showed enhanced cell membrane permeability and high human oral absorption compared to current anti-tuberculotics. Conclusions: Combined use of QSAR models that considered binding of the BHMBs to InhA, pharmacophore model, and ADME properties helped to recognize bound active conformation of the benzamide inhibitors, permitted in silico screening of VCL of compounds sharing benzamide scaffold and identification of new analogs with predicted high inhibitory potencies and favorable pharmacokinetic profiles.

## 1. Introduction

**Figure 1:**
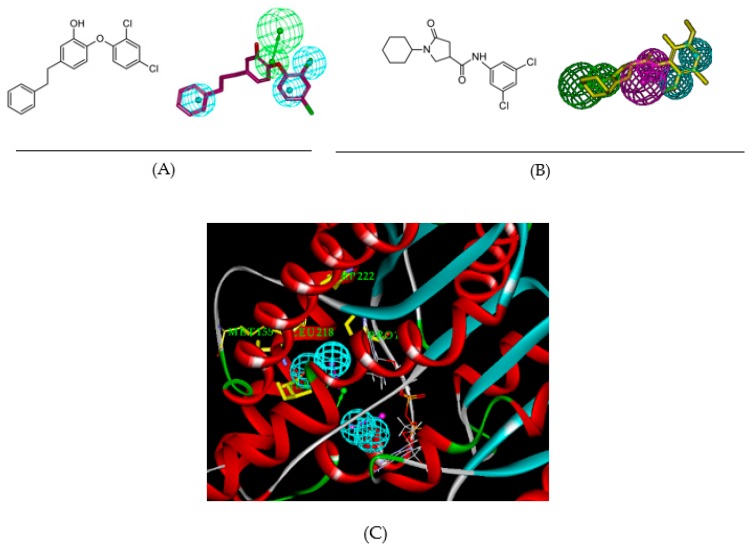
(**A**) 3D-QSAR pharmacophore model (PH4) for triclosan (TCL) derivatives displaying 3 HYD (cyan) features and the mapping of the most active analog synthesized (IC_50_ = 21 nM [16], PDB: 3FNH [21], five key interactions with InhA: HB—Tyr158, π–π—NAD and hydrophobic contacts). (**B**) PH4 for pyrrolidine carboxamide (PCAM) derivatives displaying 2 HYD (light blue) and the mapping of the most active derivative synthesized (IC_50_ = 390 nM [17], PDB: 4U0J [12], main interactions with InhA: HB—Tyr158, HB—NAD). (**C**) PH4 for the active site of InhA, depicted in ribbon, (PDB: 4DRE [22]) with one acceptor in green color, one donor in purple color, and 4 HYD features (cyan), for clarity the acceptor and donor spheres were removed. The LHP is enclosed by 2 residues in yellow color and labeled in green surrounding two HYDs.

“*United to end tuberculosis: an urgent global response to a global epidemic*” is the title of the declaration adopted by the UN General Assembly on the fight against tuberculosis (TB), which reaffirmed the commitment to end the tuberculosis epidemic globally by 2030 [1]. The General Assembly acknowledged that the Millennium Development Goals [2] and associated strategies helped to reverse the trend of the tuberculosis epidemic and until 2016 reduced the tuberculosis mortality by 37%. Nonetheless, the current WHO year report [3,4] revealed alarming statistics showing that throughout the world about 3 TB deaths occurred and about 19 persons developed TB every minute. On the other hand, drug development against *Mycobacterium tuberculosis* (*Mt*), despite the increased occurrence of multiple drug resistant (MDR-TB) and extensively drug resistant (XDR-TB) strains, has reached a noticeable progress on inhibitor design against 2-trans enoyl-acyl carrier protein reductase (InhA), the most frequently addressed validated mycobacterial drug target. The division of the InhA substrate binding site into three structural subsites (site I, catalytic; site II, hydrophobic; site III, hydrophilic) and the determination of the catalytic residue Tyr158 conformation (‘in’ or ‘out’), have paved the way for the improvement in structure-based design and development of novel InhA inhibitors [5]. Another important observation is connected with the presence of a previously unnoticed interaction pocket formed by the side chains of Phe41 and Arg43 at the InhA active site displaying Tyr158 ‘out’ conformation [6]. Promising drug candidates, which target the isoniazid-resistant *Mt*, are direct InhA inhibitors that do not require KatG (*Mycobacterium tuberculosis* catalase-peroxidase) activation [7]. Recent useful structural information involving key binding site residues identified by site-directed mutations of the InhA gene revealed that these residues (except Ser94 and Tyr158) interact with the ligand mostly through hydrophobic contacts [8]. The long list of known InhA inhibitors may be divided into, on the one hand, class 1 scaffolds: triclosan derivatives (TCL) [9], diphenyl ether [10,11], pyrrolidine carboxamide (PCAM) [12], and aryl amide derivatives [13] with Tyr158 ‘in’ conformation and typical stacking interaction with the Phe97 residue. On the other hand, class 2 scaffolds include methyl-thiazole derivatives [5], pyrazoles [14], benzamides [15] with Tyr158 ‘out’ conformation and interaction with the Phe41 and Arg43 pocket instead of the stacking with Phe97. The 3D-QSAR pharmacophores (PH4) for InhA inhibition are available for class 1 TCL and PCAM inhibitors only [16,17] but not for the class 2 compounds. Figure 1A,B show various numbers of hydrophobic features (HYD) for the PH4 of TCL and PCAM. The third HYD feature of TCL PH4 suggests that a bulky group can fill large hydrophobic pocket (LHP, site II) delimited by residues Met155, Pro193, Ile215, Leu217, Leu218, and Trp222 as a major structural requirement for efficient InhA inhibition [18]. Indeed, the best substitutions on candidates with the Triclosan scaffold direct a nonpolar group containing an ethyl linker capped by phenyl (IC_50_^exp^ = 21 nM) or pentyl group (IC_50_^exp^ = 11 nM with removal of all Cl atoms) to this LHP. The preliminary ‘interaction generation’ analysis of the InhA active site with no ligand bound (PDB: 4DRE, Figure 1C) revealed at least four HYD features, two of them located in the LHP. Structure–activity relationships involving interactions of 3D pharmacophore have been previously reported for HIV-1 inhibition, genetic disorders treatment, or proton pump inhibition [19,20,21].

The main objective of this work was to design novel potent N-benzyl-4-((heteroaryl)methyl) benzamides (BHMBs) based on a series of 19 (training set) plus 6 (validation set) nanomolar inhibitors with observed inhibitory potencies as low as IC_50_^exp^ = 20 nM [23]. Starting with in situ modification of the crystal structure of InhA-BHMB_2_ complex (PDB: 4QXM), we have elaborated a QSAR model which correlated Gibbs free energies of InhA-BHMB_x_ complex formation with the potencies IC_50_^exp^ and determined the active conformation of BHMBs bound at the active site of InhA of *Mt* (MM-PB complexation approach). Based on this active conformation we have formulated 3D QSAR pharmacophore of InhA inhibition (PH4). Large virtual library of compounds sharing the BHMB scaffold has been generated and in silico screened with the PH4. The screening yielded virtual hits that exhibited predicted inhibitory potencies IC_50_^pre^ more than 60 times lower than the most active training set compound BHMB1. Several of the identified putative inhibitors displayed favorable ADME profiles. Moreover, a series of drugs currently used in clinical practice, which include the benzamide scaffold in their molecular structure, were assessed with our new PH4 for InhA inhibition. Top five approved drugs identified by the PH4 screening exhibited predicted potencies IC_50_^pre^ ranging from 1.7 to 60 nM.

## 2. Results

### 2.1. Training and Validation Sets

The training set of 19 BHMBs and validation set of another 6 analogs (Table 1) were selected from a homogeneous series of InhA inhibitors with known experimentally determined inhibitory activities originating from a single laboratory [23]. The whole series was obtained by variations at two positions R_1_ and R_2_ of the phenyl ring and amide group as shown in Table 1. The experimental half-maximal inhibitory concentrations (20 ≤ IC_50_^exp^ ≤ 5930 nM) [23] cover a sufficiently wide concentration range for building of a reliable QSAR model. The ratio between the sizes of training and validation sets remains a critical point of correct classification but is limited by the count of the set of homologous compounds available from the literature [24].

### 2.2. QSAR Model

#### 2.2.1. One Descriptor QSAR Models

Each of the 19 training sets (TS) and 6 validation sets (VS) InhA-BHMBx complexes (Table 1), was prepared by in situ modification of the refined template crystal structure (PDB entry code 4QXM [23]) of the complex InhA-BHMB2 as described in the Methods section. Further, the relative Gibbs free energy of the InhA-BHMBx complex formation (∆∆G_com_) was computed for each of the 25 optimized enzyme–inhibitor complexes. Table 2 lists computed values of ∆∆G_com_ and its components as defined in Equation (7), for the TS and VS of benzamides [23]. The QSAR model explained variation in the BHMBs experimental inhibitory potencies (*p*IC_50_^exp^ = −*log*_10_(IC_50_^exp^) [23]) by correlating it with computed GFE ∆∆G_com_ through linear regression (Equation (8), Table 2). In addition, significant correlation obtained in this QSAR relationship permitted to determine the active bound conformation of the BHMBs at the InhA binding site and enabled definition of the PH4 pharmacophore. In search for a better insight into the binding affinity of BHBMs towards *Mt*InhA, we have analyzed the enthalpy of complexation in gas phase ∆∆H_MM_ by correlating it with the *p*IC_50_^exp^. The validity of this linear correlation (for statistical data of the regression see Table 3, Equation A) allowed assessment of the significance of inhibitor-enzyme interactions (∆∆H_MM_) when solvent effect and loss of entropy of the inhibitor upon binding to the enzyme were neglected. This correlation explained about 94% of the *p*IC_50_^exp^ data variation and underlined the role of the enthalpic contribution to the binding affinity of the ligand. Similarly, the more advanced descriptor, namely the GFE of the InhA-BHMBx complex formation including all components: ∆∆H_MM_, ∆∆TS_vib_ and ∆∆G_sol_, has been assessed (for statistical data see Table 3, Equation B). Relatively high values of the regression coefficient R^2^, leave-one-out cross-validated regression coefficient R^2^_xv_ and Fischer F-test of the correlation suggest strong relationship between the 3D model of inhibitor binding and the observed inhibitory potencies of the BHBMs [23]. Therefore, structural information derived from the 3D models of InhA-BHMBx complexes can be expected to lead to reliable prediction of InhA inhibitory potencies for new BHMBs analogs based on the QSAR model B, Table 3.

**Figure 2:**
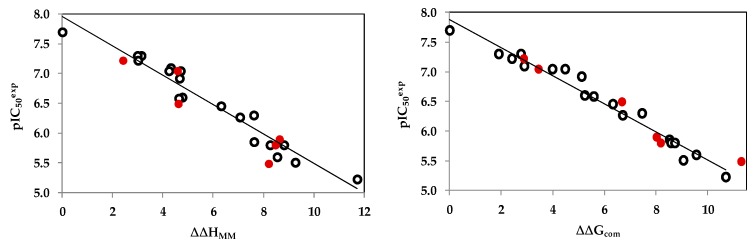
(Left) plot of correlation equation between pIC_50_^exp^ and relative enthalpic contribution to the GFE (Equation (6)) ∆∆H_MM_ [kcal⋅mol^−1^]. (Right) similar plot for relative complexation Gibbs free energies of the InhA-BHMBx complex formation ∆∆G_com_ [kcal⋅mol^−1^] of the training set [23]. The validation set data points are shown in red color.

The statistical data confirmed validity of the correlation Equations (A) and (B) plotted on Figure 2. The ratio *p*IC_50_^pre^/*p*IC_50_^exp^ ≅ 1 (the *p*IC_50_^pre^ values were estimated using correlation Equation B, Table 3) calculated for the validation set BHMV1-6 documents the substantial predictive power of the complexation QSAR model from Table 2. Thus, the regression Equation B (Table 3) and computed ∆∆G_com_ GFEs can be used for prediction of inhibitory potencies IC_50_^pre^ against *Mt*InhA for novel BHMB analogs, provided they share the same binding mode as the training set benzamides BHMB1-19.

#### 2.2.2. Binding Mode of BHMBs

**Figure 3:**
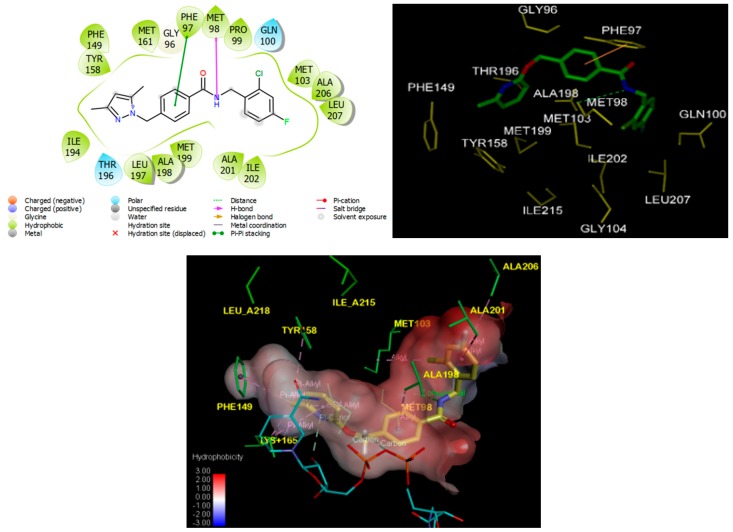
(Left) 2D schematic interaction diagram of the most potent inhibitor BHMB1 [23] at the active site of InhA of *Mt*. (Right) 3D structure of the InhA active site with bound inhibitor BHMB1. (Bottom) Hydrophobic surface of the active site of InhA showing conventional hydrogen bonds (green) and alkyl group hydrophobic contacts (pink). Surface coloring legend: red = hydrophobic, blue = hydrophilic and white = intermediate.

Structural information enzyme–inhibitor interactions retrieved from the crystal structure of InhA-BHMB1 complex [23] showed that BHMBs are InhA class 2 specific inhibitors. As indicated in Figure 3, in the catalytic site I residue Tyr158 adopted the ‘out’ conformation [5]; the pyridine moiety of the ligand (referred to as the “warhead” [6]) is in π–alkyl contact with Tyr158 and nicotinamide ring and π–donor with the hydroxyl group adjacent to the ribose of NADH. The central benzene ring of the inhibitor forms a recently observed π–π stacking interaction with Phe97 [6]. In the hydrophobic site II, the dihalide-substituted benzene ring sits in the hydrophobic substrate cavity, surrounded by side chains of predominantly nonpolar residues: Met103, Met98, Ile202, Leu207, Gly104, Phe149, Ala157, Tyr158, and Ile215 [6,23]. In the hydrophilic site III, the inhibitor makes hydrogen bonds with the sidechain of catalytic Met98, a key interaction reported recently [6]. These specific contacts were observed for the novel class of InhA inhibitors except the interaction with the “Phe41–Arg43” pocket previously unreported before Soutter et al. [6] and which will be discussed later.

### 2.3. Interaction Energy

**Figure 4:**
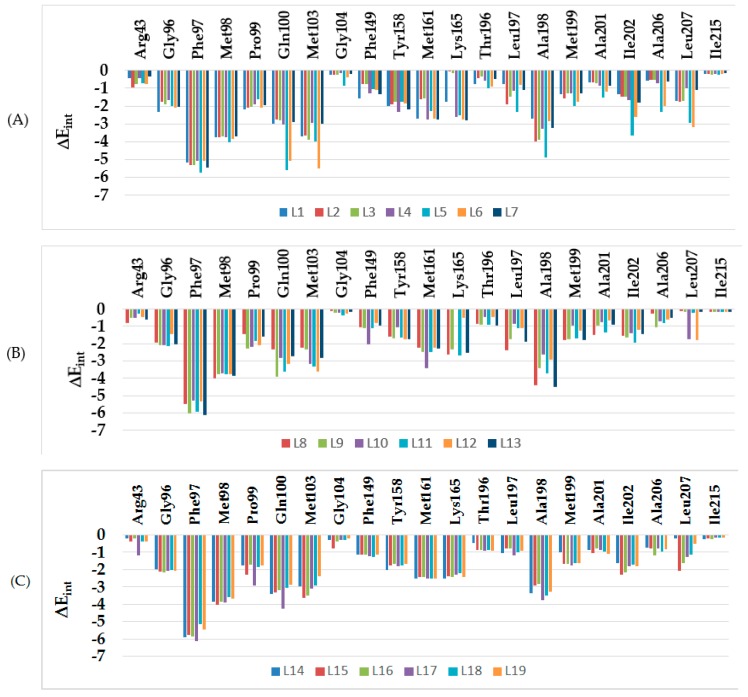
Mechanics intermolecular interaction energy E_int_ breakdown to residue contributions in [kcal.mol^−1^]: (**A**) the most active inhibitors BHMB1-7, (**B**) moderately active inhibitors BHMB8-13, (**C**) less active inhibitors BHMB14-19, Table 2 [23].

Other key structural information was provided by the interaction energy (IE, ΔE_int_) diagram obtained for each training set inhibitor. IE breakdown to contributions from InhA active site residue is helpful for the choice of relevant R_1_-groups (site I) and R_2_-groups (site II) which could improve the binding affinity of BHMB analogs to the *Mt*InhA and subsequently enhance the inhibitory potency. A comparative analysis of computed IE for training set BHMBs (Figure 4) divided into three classes (highest, moderate, and lowest activity) has been carried out to identify the residues for which the contribution to binding affinity could be increased. However, the comparative analysis showed about the same level of IE contributions from active site residues for all three classes of inhibitors. Therefore, no suggestions of suitable substitutions able to improve the binding affinity as we previously reported for thymine-like inhibitors of *Mt* thymidine monophosphate kinase design could be proposed [25]. Since specific substitutions could not be proposed, we have adopted a combinatorial approach to novel BHMB analogs design and in silico screened a virtual library of 114921 BHMB analogs with help of the PH4 pharmacophore of InhA inhibition derived from the complexation QSAR model. As we can see from the IE analysis (Figure 4), the TS and VS benzamides [23] do not show significant interaction energies with the “Phe41–Arg43” pocket.

2.4. 3D-QSAR Pharmacophore Model

#### 2.4.1. InhA Active Site Pharmacophore

The interaction generation protocol in Discovery Studio molecular modeling program [26] provides the pharmacophore features of the active site of a protein. InhA predominantly displays hydrophobic features at the active site (Figure 1C) as confirmed by previously reported works [16,17], the larger being the site II hydrophobic pocket that accommodates the substrate long alkyl chains. InhA substrate-competitive inhibitors design often exploits the pocket flexibility because of the high mobility of the Tyr158, Phe149 side chains and the substrate-binding loop (Thr196–Gly208) [27].

#### 2.4.2. Generation and Validation of 3D-QSAR Pharmacophore

InhA inhibition 3D-QSAR pharmacophore was generated from the active conformation of 19 TS BHMB1-19 and evaluated by 6 VS BHMV1-6 covering a large range of experimental activity (20–5930 nM) spanning more than two orders of magnitude. The generation process is divided into three main steps: (i) the constructive step, (ii) the subtractive step, and (iii) the optimization step [26] as described earlier [17]. During the constructive phase, BHMB1 alone was retained as the lead (since only the activity of BHMB1 fulfilled the threshold criterion, IC_50_^exp^ ≤ 2 × 20 nM), and used to generate the starting PH4 features. In the subtractive phase, compounds for which IC_50_^exp^ > 20 × 10^3.5^ nM = 63246 nM were considered inactive. Accordingly, none of the training set BHMBx was inactive and no starting PH4 features were removed. Finally, during the optimization phase, the score of the pharmacophoric hypotheses was improved. Hypotheses were scored according to errors in activity estimates from regression and complexity via a simulated annealing approach. At the end of the optimization, the top scoring 10 unique pharmacophore hypotheses were kept, all displaying five-point features. The cost values, correlation coefficients, root-mean square deviation (RMSD) values, the pharmacophore features, and the max-fit value of the top 10 ranked hypotheses (Hypo1−Hypo10) are listed in Table 4. They were selected based on significant statistical parameters, such as high correlation coefficient, low total cost, and low RMSD.

The generated pharmacophore models were then assessed for their reliability based on the calculated cost parameters ranging from 70.1 (Hypo1) to 184.7 (Hypo10). The relatively small gap between the highest and lowest cost parameter corresponds well with the homogeneity of the generated hypotheses and consistency of the TS of BHMBx. For this PH4 model, the fixed cost (45.4) is lower than the null cost (528.2) by a difference ∆ = 482.8. This difference is a major quality indicator of the PH4 predictability (∆ > 70 corresponds to an excellent chance or a probability higher than 90% that the model represents a true correlation [26]). To be statistically significant, a hypothesis has to be as close as possible to the fixed cost and as far as possible from the null cost. For the set of 10 hypotheses, the difference ∆ ≥ 343.5, which attests to the high quality of the pharmacophore model. The standard indicators such as the RMSD between the hypotheses ranged from 1.610 to 3.809, and the squared correlation coefficient (R^2^) falls to an interval from 0.97 to 0.84. The first PH4 hypothesis with the closest cost (70.1) to the fixed one (45.4) and best RMSD and R^2^ was retained for further analysis. The statistical data for the set of hypotheses (costs, RMSD, R^2^) are listed in Table 4. The configuration cost (10.63 for all hypotheses) far below 17 confirms this pharmacophore as a reasonable one.

The link between the 98% significance and the number 49 scrambled runs of each hypothesis is based on the formula S = [1 − (1 + X)/Y] × 100, with X the total number of hypotheses having a total cost lower than the original hypothesis (Hypo 1) and Y the total number of HypoGen runs (initial + random runs): X = 0 and Y = (1 + 49), hence 98% = {1 − [(1 + 0)/(49 + 1)]} × 100.

**Figure 5:**
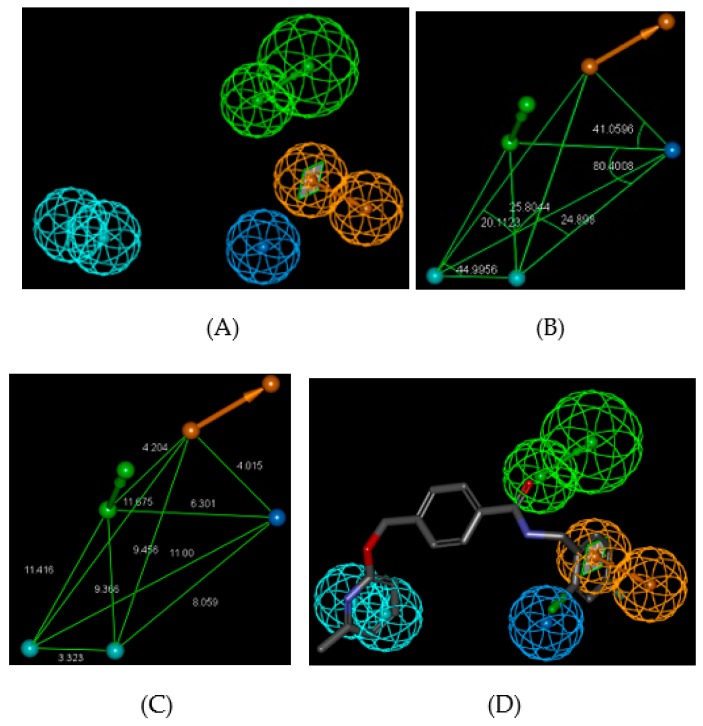

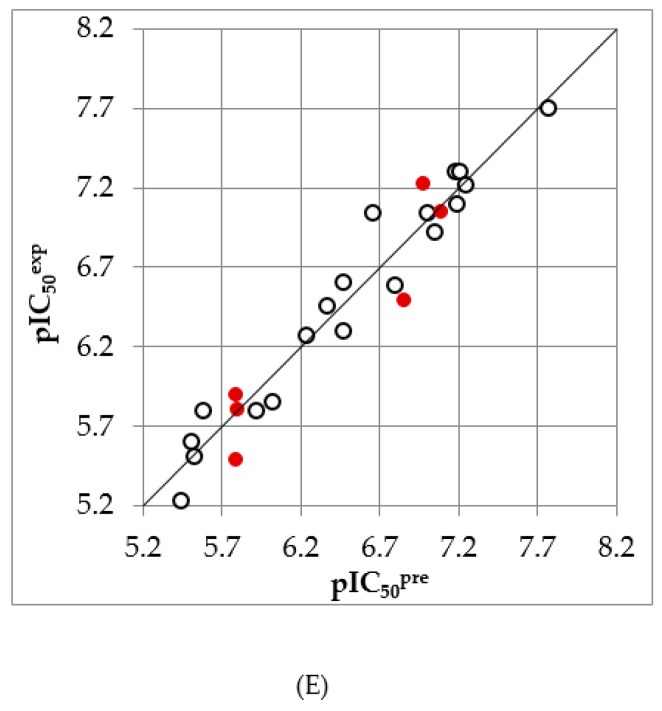
Features (**A**) coordinates of centers, (**B**) angles between centers of pharmacophoric features, (**C**) distances between centers, (**D**) mapping of pharmacophore of InhA inhibitor with the most potent molecule BHMB1. The R_1_ position in the BHMBs of TS compounds is occupied by benzene ring (except BHMB8), therefore the second HYD expected from the InhA active site PH4 (Figure 1C) is replaced by Ar feature in BHMBs PH4. Feature legend: HYDA = Hydrophobic Aliphatic (blue), HYD = Hydrophobic (cyan), Ar = Ring aromatic (orange), HBA = Hydrogen bond Acceptor (green). (**E**) correlation plot of experimental vs. predicted inhibitory activity (open circles correspond to TS, red dots to VS).

The evaluation of Hypo 1 was performed first through Fischer’s randomization cross-validation test. The CatScramble program was used to randomize the experimental activities of the training set. At 98% confidence level, each of the 49 scramble runs created ten valid hypotheses, using the same features and parameters as in the generation of the original 10 pharmacophore hypotheses. Among them, the cost value of Hypo1 is the lowest compared with those of the 49 randomly generated hypotheses, as we can see in Table 4 where the lowest cost of the 49 random runs is listed for each original hypothesis, and none of them was as predictive as the original hypotheses generated shown in Table 4. Thus, there is a 98% probability that the best selected hypothesis Hypo1 represents a pharmacophore model for inhibitory activity of BHMBs with a similar level of predictive power as the complexation QSAR model, which relies on the BHMBx active conformation from 3D structures of the InhA-BHMBx complexes and computed GFE of enzyme–inhibitor binding ΔΔG_com_. Another evaluation of Hypo 1 is the mapping of the best active training set BHMB1 (Figure 5) displaying the geometry of the Hypo1 pharmacophore of InhA inhibition. The regression equation for *p*IC_50_^exp^ vs. *p*IC_50_^pre^ estimated from Hypo1: *p*IC_50_^exp^ = 1.0013 × *p*IC_50_^pre^ − 0.0085 (n = 19, R^2^ = 0.95, R_xv_^2^ = 0.93, F-test = 324.25, σ = 0.165, α > 98 %) is also plotted on Figure 5.

We can carry out computational design and selection of new BHMB analogs with elevated inhibitory potencies against *Mt*InhA, based on a strategy using the noticeable presence of the hydrophobic features included in the best pharmacophore model at the position of R_2_ coupled with mapping of R_1_ to the aromatic ring feature and the appropriate ring substitution to the hydrophobic aliphatic feature in Hypo1 (Figure 5).

### 2.5. Virtual Screnning

In silico screening of a virtual (combinatorial) library can lead to hit identification as it was shown in our previous works on inhibitors design [17,28,29].

#### 2.5.1. Virtual Library

An initial virtual library (VL) was generated by substitutions at positions for R_1_ and R_2_ (see Table 5) on the benzamide scaffold. During the virtual library enumeration, all 339 R-groups listed in Table 5 were attached to in positions R_1_ and R_2_ of the BHMB scaffold to form a combinatorial library of the size: R_1_ × R_2_ = 339 × 339 = 114,921 analogs. This initial diversity library was generated from building blocks (chemicals) listed in the databases of available chemicals [30]. To design a more focused library of a reduced size and increased content of drug-like molecules, we have introduced a set of filters and penalties such as the Lipinski rule-of-five [31], which helped to select a smaller number of suitable BHMBs that could be submitted to in silico screening. This focusing has reduced the size of the initial library to 73,565 analogs, 64% of its original number size.

#### 2.5.2. In Silico Screening of Library of BHMBs

The focused library of 73,565 analogs was further screened for molecular structures matching the 3D-QSAR PH4 pharmacophore model Hypo1 of InhA inhibition. 238 BHMBs mapped to at least 2 pharmacophoric features, 90 of which mapped to at least 4 features of the pharmacophore. These best fitting analogs (PH4 hits) then underwent complexation QSAR model screening. The computed GFE of InhA-BHMBx complex formation, their components, and predicted half-maximal inhibitory concentrations IC_50_^pre^ calculated from the correlation Equation B (Table 3) are listed in Table 6.

### 2.6. Novel BHMB Analogs

**Figure 6:**
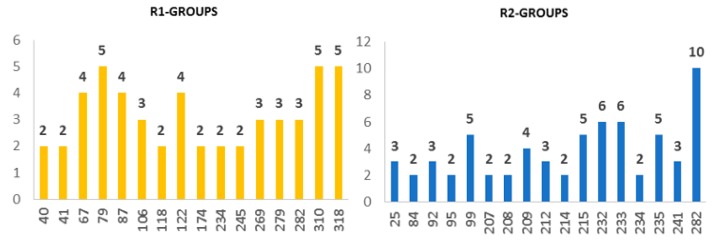
Histograms of frequency of occurrence of individual R-groups in the 90 best selected analogs mapping to four features of the PH4 pharmacophore hypothesis Hypo1 (for the structures of the fragments see Table 5); R_1_ = -3-Br-2-(thiazol-2-yl)propyl (79); -3-F-1H-pyrazol-1-yl (310); -5-Et-1H- pyrazol-1-yl (318) and R_2_ = -(3-F-cyclopenta-2,4-dien-1-yl)(MeAmino)Me (232); -(2-F-cyclopenta-2, 4-dien-1-yl)(MeAmino)Me (233) and -Bz-2-Me (282).

The design of virtual library of novel analogs was guided by structural information retrieved from the BHMBx active conformation and was used for the selection of appropriate substituents (R_1_- and R_2_-groups). In order to identify which substituents lead to new inhibitor candidates with the highest predicted potencies towards the InhA of *Mt*, we have prepared histograms of the frequency of occurrence of R_1_- and R_2_-groups among the 90 best fit PH4 hits (Figure 6). The histograms show that the R_1_ groups 79, 310, and 318 were represented with the highest frequency of occurrence (5) among the 90 BHMB hits. The R_2_-groups most frequently represented in this subset are 282 (10) and 232, 233 (6) and 99, 215 and 235 (5). The top ten scoring virtual hits, namely, analogs are 79-92 (IC_50_^pre^ = 33 pM), 79-339 (39 pM), 79-338 (47 pM), 41-93 (52 pM), 234-99 (90 pM), 238-25 (90 pM), 122-92 (100 pM), 220-99 (110 pM), 310-99 (110 pM), and 166-92 (150 pM). They include the following substituents at R_1_ position: 79: 3-Br-2-(thiazol-2-yl)Pr (3), 41: Thiophen-2-ylMe (1), 234: (2,3-diFC_5_H_7_- 2,4-dien-1-yl)(mercapto-amino)Me (1), 238: FNH(3-FC_5_H_7_-2,4-dien-1-yl)Me (1), 122: 2-(F_3_C)Pyri- din-3-yl (1), 220: 2,3-diBr C_5_H_7_-2,4-diene-carbonyl (1), 310: 3-F-1-H-pyrazol-1-yl (1), 166: 4-(3-NH_2_-1- H-pyrazol-1-yl)Ph (1), or at R_2_ position: 92: 4-HO-Hexyl (3), 339: 2-(thiazol-2-yl)butyl (1), 338: 2-(thiazol-2-yl)pentyl (1), 93: 5-NH_2_-6-Br Hexyl (1), 99: 6-NH_2_Octyl (3), 25: 6-MeOctyl (1). Due to amino acid composition of the larger hydrophobic pocket, the R_2_-groups display preferences for larger aliphatic building blocks from butyl to octyl [5].

**Figure 7:**
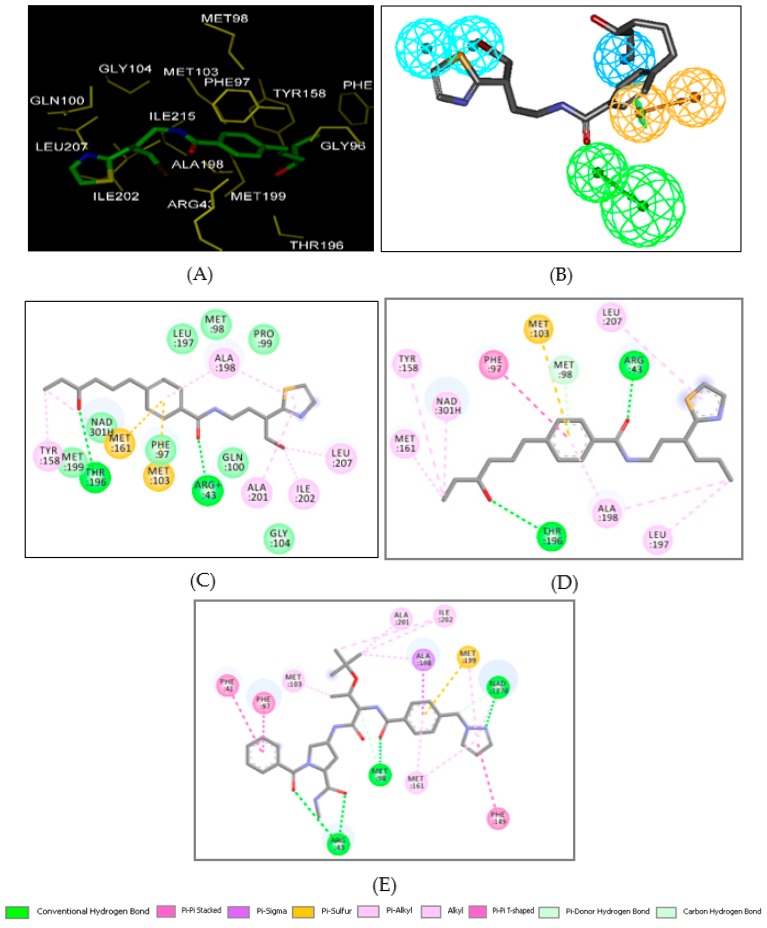
(**A**) Close up of virtual hit 79–92, the most active designed BHMB analog (IC_50_^pre^ = 33 pM) at the active site of InhA. Interacting residues are colored yellow, and NADH is not shown for clarity. (**B**) Mapping of the BHMB 79–92 to InhA inhibition pharmacophore. (**C**) 2D schematic interaction diagram of the BHMB 79–92 at the active site of *Mt*InhA. (**D**) 2D schematic interaction diagram of the analog BHMB 79–339 (IC_50_^pre^ = 39 pM) at the active site of *Mt*InhA. (**E**) 2D schematic interaction diagram of the ligand ((2S,4S)-N-methyl-4-[[(2S,3R)-3-[(2-methylpropan-2-yl)oxy]-2-[[4-(pyrazol-1- ylmethyl)phenyl]carbonylamino]butanoyl]amino]-1-(phenylcarbonyl)pyrrolidine-2-carboxamide) in complex with *Mt*InhA (PDB: 5G0W) displaying the HB contact with Arg43 as reported in [6].

**Figure 8:**
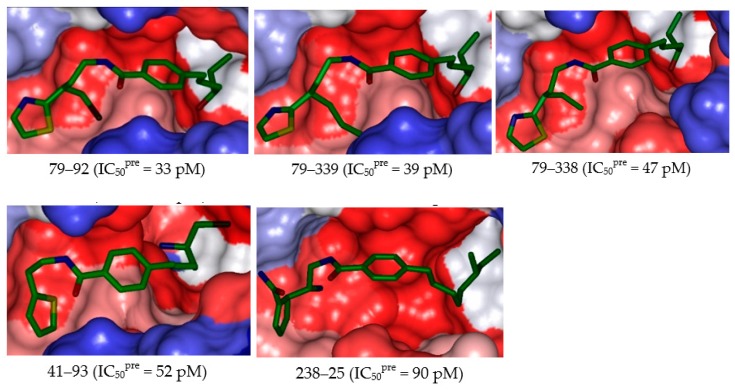
Surface of the active site of *Mt*InhA with bound 5 best active designed BHMB analogs. The binding site surface is colored according to residue hydrophobicity: red = hydrophobic, blue = hydrophilic, and white = intermediate.

The substitutions in R_1_ and R_2_ positions of BHMBs led to an overall increase of affinity of InhA binding as exemplified by the inhibitory potencies of majority of new designed analogs. The best designed benzamide BHMB 79–92 displays predicted half-minimal inhibitory concentration of IC_50_^pre^ = 33 pM that is about 61 times lower than that of the most active compound of the TS, namely the BHMB1 with IC_50_^exp^ = 20 nM, Figure 7 and Figure 8.

### 2.7. Pharmacokinetic Profile of Novel BHMB Analogs

The pharmacokinetic profile of InhA inhibitors remains an important issue [7]. The best designed triclosan derivative with very low oral bioavailability due to its poor water solubility and rapid phase II metabolism has to be optimized for a possible use as antituberculotic and eventually antimalarial in case of high affinity toward *Pf*EACP [32]. Among the ADME-related properties displayed in Table 7, such as octanol-water partitioning coefficient; aqueous solubility; blood-brain partition coefficient; Caco-2 cell permeability; serum protein binding; number of likely metabolic reactions; and another eighteen descriptors related to absorption, distribution, metabolism, and excretion (ADME) of the new analogs, were computed by the QikProp program [33] based on the method of Jorgensen [34,35]. Experimental data from more than 710 compounds including about 500 drugs and related heterocycles were used to produce regression equations correlating experimental and computed descriptors, resulting in an accurate prediction of pharmacokinetic properties of molecules. In line with the observed unfavorable pharmacokinetic exposure for the best active triclosan derivative, the predicted oral bioavailability of novel BHMB analogs ranges from 86% to 100%. Since a value greater than 80% is considered good, ten analogs among the best predicted 14 display a human oral absorption in gastrointestinal tract (HOA) of 100%. Drug likeness (#stars)—the number of property descriptors that fall outside the range of optimal values determined for 95% of known drugs out of 24 selected descriptors computed by the QikProp—was used as an additional ADME-related compound selection criterion. The values for the best active designed BHMBs are compared with those computed for drugs used for treatment of tuberculosis or currently undergoing clinical trials (Table 7). Our best designed analogs all display #stars equal to zero, meaning that the optimal value range of none of the drug-likeness descriptors was violated. Thus the designed BHMBs display a favorable pharmacokinetic profile.

### 2.8. Predicted InhA Inhibition Potency for Current Drugs Bearing Benzamide Scaffold

**Figure 9:**
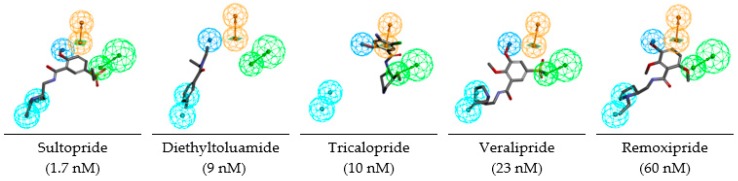
Mapping of the benzamide drugs currently used in clinical practice to the InhA inhibition pharmacophore. Feature legend: HYDA = Hydrophobic Aliphatic (blue), HYD = Hydrophobic (cyan), Ar = Ring aromatic (orange), HBA = Hydrogen bond Acceptor (green). Only the top five predicted IC_50_^pre^ are displayed.

Since the benzamide scaffold has been analyzed in this study, drugs currently used in the clinical practice sharing this scaffold are worth evaluating with the help of our 3D-QSAR generated PH4 pharmacophore. The list of 24 compounds given in Table 8 is mostly indicated for treatment of neural disorders and cancers [36]. As we can see on Figure 9, the mapping of the five most potent predicted *Mt*InhA inhibitors to PH4 pharmacophore sheds light on their affinity towards the enzyme and suggests their experimental evaluation as antituberculotics as they predominantly occupy the large hydrophobic pocket. The best predicted, Sultopride (IC_50_^pre^ = 1.7 nM) is an approved drug, used in Japan, Hong Kong, and Europe for treatment of schizophrenia [36].

## 3. Discussions

### 3.1. Binding Mode of BHMBs

**Figure 10:**
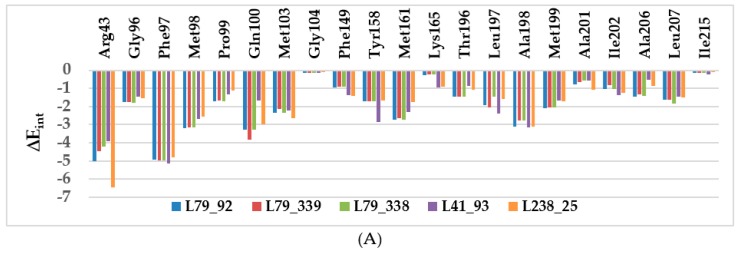

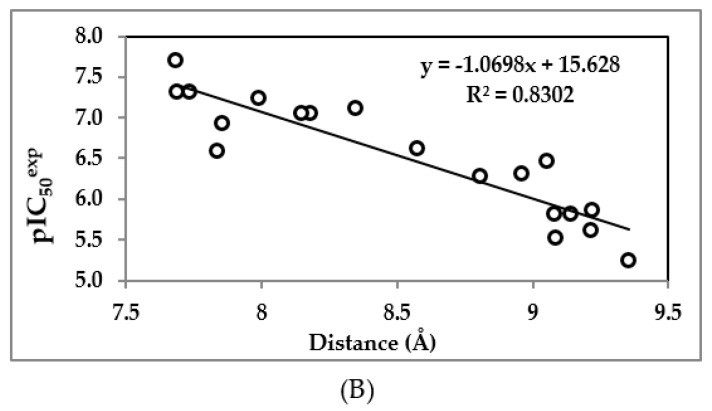
Molecular mechanics inter-molecular interaction energy E_int_ break-down to active site residue contributions in [kcal.mol^−1^]: (**A**) designed best five novel BHMB analogs (the color coding refers to ligands given in the legend), (**B**) correlation between *p*IC_50_^exp^ and distance from the benzamide carbonyl oxygen to the Arg43 warhead carbon atom (7.5–9.5 Å).

To date, the most comprehensive study on InhA inhibition has been reported by Soutter et al. [6]. An important output of their DNA-encoded screening is the novel binding mode of inhibitors at the *Mt*InhA active site, where the ligand, in addition to the typical stacking contact with Phe97 reaches a pocket surrounded by Phe41 and Arg43 side chains. This results in either a π–cation or a conventional hydrogen-bond contact. According to our analysis of the InhA-BHMBx complexes of most potent inhibitors, this interaction plays a key role in the significant improvement of predicted inhibitory potencies of novel benzamides. The enzyme–inhibitor interaction energy (E_int_) breakdown to InhA active site residues contribution of best designed BHMB analogs (Figure 10) shows that in contrast to the TS and VS inhibitors [23] (Figure 4), while the contribution of Phe97 to E_int_ is almost the same, the impact of interaction with polar Arg43 increased by at least 4 kcal·mol^−1^. Surprisingly, the novel benzamide inhibitors display noticeable interaction with Phe97 and Arg43, suggesting that their length, which is comparable to that of TS BHMBs, is sufficient for concomitant interactions. Recently, site exclusion was observed for the ligands in complex with InhA (a pyrrolidine core and three substituents: benzoyl and 1-t-butoxy ethyl groups directly connected to the pyrrolidine core and a pyrazole benzaldehyde connected via the t-butoxy ethyl) benzoyl π–π stacks Phe97, methyl amide HB with Arg43), (PDB: 5G0W) [6]. As displayed in Figure 10, distance between the benzamide carbonyl oxygen and the Arg43 sidechain warhead carbon atom is relatively high for the training set compounds (7.5–9.5 Å). Interestingly, the plot of this distance vs. inhibitory activity *p*IC_50_^exp^ displays a correlation. This distance seems to be one of the key determinants for binding affinity for the class 2 InhA direct inhibitors instead of the existence of a HB. This structural specificity that emerged during the PH4 screening of the virtual library of benzamide analogs led to identification of new hits, the best of which are capable of forming a HB with the Arg43 residue.

### 3.2. PH4-Based Screening of Approved Drugs Containing Benzamide Scaffold

A library of 24 benzamide scaffold drugs from DrugBank [36] was screened with the class 2 InhA inhibition PH4. These drugs are mostly dedicated to a variety of neurological and psychiatric disorders or nausea and vomiting treatment. The top five best PH4 hits have been evaluated also with the complexation QSAR model Equation B, Table 3, to predict their antituberculotic IC_50_^pre^ towards the *Mt*InhA: Sultopride (IC_50_^pre^ = 1.7 nM), Diethyltoluamide (9 nM), Tricalopride (10 nM), Veralipride (23 nM) and Remoxipride (60 nM). Their mapping to the pharmacophore features is displayed in Figure 9. Therefore, experimental testing of these five approved drugs for *Mt*InhA inhibition and perhaps also for antibacterial effect against the *Mt* would be worthwhile.

## 4. Materials and Methods

### 4.1. Training and Validation Sets

Chemical structures and biological activities (IC_50_^exp^) of training and validation sets of *N*-benzyl-4-((heteroaryl)methyl) benzamides inhibitors of InhA used in this study were taken from literature [23]. The potencies of these compounds cover a sufficiently broad range of half-maximal inhibitory concentrations (20 ≤ IC_50_^exp^ ≤ 5930 nM) to allow construction of a QSAR model. The training set (TS) containing 19 BHMB inhibitors and the validation set (VS) including 6 BHMBs were taken from the ref. [23].

### 4.2. Model Building

Three dimensional (3D) molecular models of enzyme–inhibitor (E-I) complexes *Mt*InhA-BHMBx, free enzyme InhA and free inhibitors BHMBx were prepared from high-resolution (2.2 Å) crystal structure of a reference complex containing the training set compound *N*-(2-chloro-4-fluorobenzyl)-4-((3,5-dimethyl-1-*H*-pyrazol-1-yl)methyl)benzamide (BHMB2, Table 1) bound to the mycobacterial InhA (Protein Data Bank [37] entry code 4QXM [23]) using Insight-II molecular modeling program [38].

The structures of InhA and the E-I complexes were considered to be at pH of 7 with neutral N- and C-terminal residues and all protonizable and ionizable residues charged. No crystallographic water molecules were included in the model. The inhibitors were built into the reference structure 4QXM [23] by in situ replacing of derivatized groups in the molecular scaffold of the template inhibitor BHMB2. An exhaustive conformational search over all rotatable bonds of the replacing function groups coupled with a careful gradual energy-minimization of the modified inhibitor and active site residues of the InhA located in the vicinity of the inhibitor (within 5 Å distance) was employed to identify low-energy bound conformations of the modified inhibitor. The resulting low-energy structures of the E-I complexes were then carefully refined by minimization of the whole complex. This procedure has been successfully used for model building of viral, bacterial, and protozoal enzyme–inhibitor complexes and design of peptidomimetic, hydroxynaphthoic, thymidine, triclosan, pyrrolidine carboxamide, nitriles, and chalcone-based inhibitors [16,17,29,39,40,41,42,43,44,45,46,47].

### 4.3. Molecular Mechanics

Modeling of inhibitors, InhA, and E-I complexes was carried out by molecular mechanics using CFF91 force field [48] as described earlier [17].

### 4.4. Conformational Search

Free inhibitor conformations were derived from their bound conformations in the E-I complexes by gradual relaxation to the nearest local energy minimum as described earlier [17].

### 4.5. Solvation Gibbs Free Energies

The electrostatic component of solvation Gibbs free energy (GFE) that includes also the effects of ionic strength via solving nonlinear Poisson–Boltzmann equation [49,50] was computed by the DelPhi module in Discovery Studio [26] as described earlier [17].

### 4.6. Calculation of Binding Affinity and QSAR Model

The calculation of binding affinity expressed as complexation GFE has been described fully earlier [17].

### 4.7. Interaction Energy

The calculation of MM interaction energy (E_int_) between enzyme residues and the inhibitor CFF91 force field [48] was performed as described earlier [17].

### 4.8. Pharmacophore Generation

Bound conformations of inhibitors taken from the models of E-I complexes were used for constructing of 3D-QSAR pharmacophore (PH4) by means of Catalyst HypoGen algorithm [51] implemented in Discovery Studio [26] as described earlier [17].

### 4.9. ADME Properties

The pharmacokinetics profile of BHMBs were computed by the QikProp program [33] as described earlier [17].

### 4.10. Virtual Library Generation

The virtual library generation was performed as described earlier [17].

### 4.11. ADME-Based Library Searching

The drug-likeness selection criterion served to focus the initial virtual library as described earlier [17].

### 4.12. Pharmacophore-Based Library Searching

The pharmacophore model (PH4) described in Section 4.8 and derived from the bound conformations of BHMBs at the active site of InhA served as library searching tool as described earlier [17].

### 4.13. Inhibitory Potency Prediction

The conformer with the best mapping on the PH4 pharmacophore in each cluster of the focused library subset was used for ΔΔG_com_ calculation and IC_50_^pre^ estimation (virtual screening) by the complexation QSAR model as described earlier [17].

## 5. Conclusions

In this work novel class 2 InhA direct inhibitors with benzamide scaffold have been designed to reach the picomolar inhibitory concentration range of the predicted IC_50_^pre^ (Table 6, Figure 8). Even though these predicted inhibitory potencies may be somewhat too optimistic, they suggest that benzamide-type *Mt*InhA inhibitors more potent than the known TS and VS analogs [23] may exist. Our QSAR model provided bound InhA inhibitor conformation, from which the enzyme–inhibitor interaction energy breakdown to active site residue contribution clearly revealed structural determinants needed for binding improvement involving the favorable contacts in the three active site subsites (I, II, and III) including combination of π–π stacking with Phe97 and the HB contacts with Arg43. The derived 3D QSAR pharmacophores identified during chemical space exploration around R_1_ and R_2_ positions novel BHMBs analogs with predicted picomolar *Mt*InhA inhibitory potencies 79–92 (IC_50_^pre^ = 33 pM), 79–39 (39 pM) and 79–338 (47 pM) all display also favorable pharmacokinetic profiles compared to current antituberculotics. We believe that they are worth synthesizing and evaluating.

Moreover, current drugs sharing the benzamide scaffold have been in silico evaluated and the top five predicted drugs, Sultopride (IC_50_^pre^ = 1.7 nM), Diethyltoluamide (9 nM), Tricalopride (10 nM), Veralipride (23 nM), and Remoxipride (60 nM) are also suggested for biological evaluation as potential antituberculotics.

## Figures and Tables

**Table 1:**
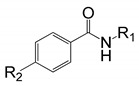
Set (BHBM1-19) and validation set (BHBV1-6) of InhA inhibitors [23] used in the preparation of QSAR models of inhibitor binding. The R_1_ and R_2_ groups are numbered in the first part of the Table as #R ≡ group index.

**#R**	**1**	**2**	**3**	**4**	**5**	**6**	**7**	**8**
R group	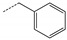	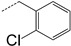	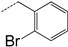	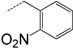	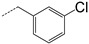	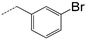	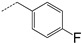	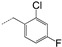
**#R**	**9**	**10**	**11**	**12**	**13**	**14**	**15**	**16**
R group	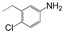	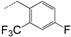	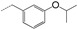	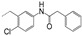	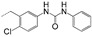	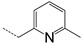	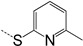	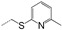
**#R**	**17**	**18**	**19**	**20**	**21**	**22**	**23**	**24**
R group	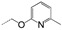	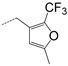	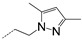	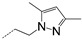	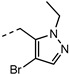	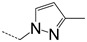	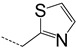	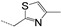
**#R**	**25**	**26**						
R group	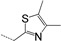	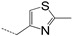						
**Training Set**	BHMB1	BHMB2	BHMB3	BHMB4	BHMB5	BHMB6	BHMB7	BHMB8
**#R_1_–#R_2_**	8–17	8–19	**8**–14	8–15	12–19	13–19	8–26	18–19
IC_50_^exp^ (nM)	20	50	50	60	80	90	90	120
**Training Set**	BHMB9	BHMB10	BHMB11	BHMB12	BHMB13	BHMB14	BHMB15	BHMB16
**#R_1_–#R_2_**	10–19	8–16	9–19	8–22	3–19	8–25	6–19	5–19
IC_50_^exp^ (nM)	250	260	350	500	540	1400	1580	1580
**Training Set**	BHMB17	BHMB18	BHMB19					
**#R_1_–#R_2_**	4–19	7–19	1–19					
IC_50_^exp^ (nM)	2510	3100	5930					
**Validation Set**	BHMV1	BHMV2	BHMV3	BHMV4	BHMV5	BHMV6		
**#R_1_–#R_2_**	8–24	21–19	8–20	2–19	11–19	8–23		
IC_50_^exp^ (nM)	60	90	320	1260	1580	3250		

**Table 2:** Gibbs free energy (binding affinity) and its components for the training set of InhA inhibitors BHMB1-19 and validation set inhibitors BHMV1-6 [23].

**Training Set ^a^**	**M_w_^b^**	**∆∆H_MM_^c^**	**∆∆G_sol_^d^**	**ΔΔTS_vib_^e^**	**∆∆G_com_^f^**	**IC_50_^exp g^**
	**[g⋅mol^−1^]**	**[kcal⋅mol^−1^]**	**[kcal⋅mol^−1^]**	**[kcal⋅mol^−1^]**	**[kcal⋅mol^−1^]**	**[nM]**
BHMB1	385	0	0	0	0	20
BHMB2	372	3.14	−1.75	−0.51	1.90	50
BHMB3	369	2.99	−0.56	−0.33	2.76	50
BHMB4	387	3.01	−3.09	−2.50	2.41	60
BHMB5	487	4.31	−5.38	−3.96	2.89	80
BHMB6	488	4.70	−1.02	−0.30	3.98	90
BHMB7	377	4.24	−3.22	−3.45	4.47	90
BHMB8	391	4.66	−0.01	−0.46	5.11	120
BHMB9	405	4.77	0.64	0.18	5.23	250
BHMB10	401	4.63	0.33	−0.62	5.58	260
BHMB11	369	6.31	1.59	1.58	6.32	350
BHMB12	358	7.61	−1.80	−1.63	7.45	500
BHMB13	398	7.06	0.29	0.65	6.70	540
BHMB14	389	7.62	−1.31	−2.20	8.51	1400
BHMB15	398	8.81	0.77	1.00	8.57	1580
BHMB16	354	8.26	1.50	1.04	8.72	1580
BHMB17	364	8.54	2.95	1.94	9.55	2510
BHMB18	337	9.26	1.80	2.01	9.05	3100
BHMB19	319	11.72	1.81	2.84	10.69	5930
**Validation Set**	**M_w_^b^**	**∆∆H_MM_^c^**	**∆∆G_sol_^d^**	**ΔΔTS_vib_^e^**	**∆∆G_com_^f^**	***p*IC_50_^pre^/*p*IC_50_^exp h^**
	**[g⋅mol^−1^]**	**[kcal⋅mol^−1^]**	**[kcal⋅mol^−1^]**	**[kcal⋅mol^−1^]**	**[kcal⋅mol^−1^]**	
BHMV1	375	2.41	−2.49	−2.95	2.87	0.99
BHMV2	416	4.58	0.32	1.45	3.44	1.00
BHMV3	386	4.61	4.53	2.47	6.67	0.97
BHMV4	354	8.63	1.49	2.12	8.01	1.01
BHMV5	377	8.47	0.47	0.77	8.17	1.02
BHMV6	361	8.20	0.48	−2.62	11.29	0.95

^a^ for the chemical structures of the training set of inhibitors see Table 1; ^b^ M_w_ is the molar mass of inhibitors; ^c^ ΔΔH_MM_ is the relative enthalpic contribution to the GFE change related to E-I complex formation derived by MM; ΔΔH_MM_ ≈ [E_MM_{E-I_x_} − E_MM_{I_x_}] − [E_MM_{E-I_ref_} − E_MM_{I_ref_}], I_ref_ is the reference inhibitor BHMB1; ^d^ ΔΔG_sol_ is the relative solvent effect contribution to the GFE change of E-I complex formation: ΔΔG_sol_ = [G_sol_{E-I_x_} − G_sol_{I_x_}] − [G_sol_{E-I_ref_} − G_sol_{I_ref_}]; ^e^ −ΔΔTS_vib_ is the relative entropic contribution of inhibitor I_x_ to the GFE of E-I_x_ complex formation: ΔΔTS_vib_ = [TS_vib_{I_x_}_E_ − TS_vib_{I_x_}] − [TS_vib_{I_ref_}_E_ − TS_vib_{I_ref_}]; ^f^ ΔΔG_com_ is the overall relative GFE change of E-I_x_ complex formation: ΔΔG_com_ ≈ ΔΔH_MM_ + ΔΔG_sol_ − ΔΔTS_vib_; ^g^ IC_50_^exp^ is the experimental half-maximal inhibition concentration of InhA obtained from ref. [23]; ^h^ ratio of predicted and experimental half-maximal inhibition concentrations *p*IC_50_^pre^/*p*IC_50_^exp^ (*p*IC_50_^pre^ = −log_10_IC_50_^pre^) was predicted from computed ΔΔG_com_ using the regression equation for InhA shown in Table 3, B.

**Table 3:** Analysis of computed binding affinities ∆∆G_com_, its enthalpic component ∆∆H_MM_, and experimental half-maximal inhibitory concentrations pIC_50_^exp^ = −log_10_IC_50_^exp^ of BHMBs towards *Mt*InhA [23].

Statistical Data of Linear Regression	(A)	(B)
*p*IC_50_^exp^ = −0.2465 × ∆∆H_MM_ + 7.9554 (A)		
*p*IC_50_^exp^ = −0.2370 × ∆∆G_com_ + 7.8783 (B)		
Number of compounds n	19	19
Squared correlation coefficient of regression R^2^	0.94	0.97
LOO cross-validated squared correlation coefficient R^2^_xv_	0.92	0.95
Standard error of regression σ	0.178	0.135
Statistical significance of regression, Fisher F-test	274.92	493.24
Level of statistical significance α	>95 %	>95 %
Range of activities IC_50_^exp^ [nM]	20–5930	

**Table 4:** Parameters of 10 generated PH4 pharmacophoric hypotheses for InhA inhibitor after Cat-Scramble validation procedure (49 scrambled runs for each hypothesis at the selected level of confidence of 98%).

Hypothesis	RMSD ^a^	R^2 b^	Total Costs ^c^	Costs Difference ^d^	Closest Random ^e^
Hypo1	1.610	0.97	70.1	458.1	147.15
Hypo2	1.973	0.96	82.6	445.6	155.68
Hypo3	2.281	0.95	94.8	433.4	183.64
Hypo4	2.673	0.93	114.3	413.9	201.42
Hypo5	2.751	0.92	118.9	409.3	205.69
Hypo6	2.916	0.91	126.3	401.9	213.65
Hypo7	3.396	0.88	155.5	372.8	214.63
Hypo8	3.586	0.87	169.1	359.2	241.58
Hypo9	3.709	0.86	177.2	351.1	247.34
Hypo10	3.809	0.85	184.7	343.5	260.41

^a^ root mean square deviation; ^b^ squared correlation coefficient; ^c^ overall cost parameter of the PH4 pharmacophore; ^d^ cost difference between Null cost and hypothesis total cost; ^e^ lowest cost from 49 scrambled runs at a selected level of confidence of 98%. The Fixed Cost = 45.4 with RMSD = 0, the Null Cost = 528.2 with RMSD = 7.215 and the Configuration cost = 10.63.

**Table 5:**
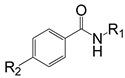
R_1_- and R_2_-groups (fragments, building blocks, substituents) used in the design of the initial diversity virtual combinatorial library of benzamides.

R-groups ^a^					
1	Me	2	1-ClMe	3	Et
4	1-F-Et	5	propyl	6	1-Brpropyl
7	Me-thiol	8	1-BrMe	9	1-ClEt
10	1-F-propyl	11	butyl	12	1-F-Me
13	1-BrEt	14	1-Clpropyl	15	1-F-butyl
16	1-Clbutyl	17	1-F-pentyl	18	hexyl
19	1-Brhexyl	20	isopentyl	21	6,6-diMeheptyl
22	6-diMe-heptyl	23	3,3-diMe-butyl	24	3-Mepentyl
25	6-Meoctyl	26	3-Etpentyl	27	Me-C_3_H_5_
28	Butyl-C_3_H_5_	29	Me-C_4_H_7_	30	Butyl-C_4_H_7_
31	Me-C_5_H_9_	32	Butyl-C_5_H_9_	33	Me-C_6_H_11_
34	Butyl-C_6_H_11_	35	cycloprop-2-en-1-yl	36	Thiophen-2-yl
37	Thiophen-3-yl	38	Furan-2-yl	39	5-Me-thiophen-2-yl
40	3,4,5-triMe-thiophen-2-yl	41	Thiophen-2-ylMe	42	furan-3-ylMe
43	3-Me-thiophen-2-yl	44	3,5-diMe-thiophen-2-yl	45	Furan-2-ylMe
46	2-(thiophen-2-yl)Et	47	4-Me-thiophen-2-yl	48	4,5-diMe-thiophen-2-yl
49	2-(furan-2-yl)Et	50	Thiophen-3-ylMe	51	Ph
52	4-ClPh	53	4-F-Ph	54	4-BrPh
55	p-tolyl	56	3-F-Ph	57	3-ClPh
58	3-BrPh	59	m-tolyl	60	2-F-Ph
61	2-ClPh	62	2-BrPh	63	o-tolyl
64	4-OHPh	65	4-MeOPh	66	4-OH-Bn
67	4-MeO-Bn	68	-Bn	69	4-F-Bn
70	4-Cl-Bn	71	4-Br-Bn	72	4-Me-Bn
73	3,5-diMe-Bn	74	4-((1H-imidazol-2-yl)Me)-Bn	75	aminoMe
76	diClMe	77	2-(1H-imidazol-2-yl)Et	78	2-Clpropyl
79	3-Br-2-(thiazol-2-yl)propyl	80	(Furan-3-ylMe)thio	81	diBrMe
82	2-amino-2-ClEt	83	3,3-diBr-3-F-propyl	84	4-(pyridin-3-yl)butyl
85	F-Me-Cl	86	2-(1,3,4-thiadiazol-2-yl)Et	87	2-Br-2-(1,3,4-thiadiazol-2-yl)Et
88	1-Br-3-Clpropyl	89	4-(1H-imidazol-2-yl)butyl	90	4-Cl-3-OHbutyl
91	3-((F-Me)amino)propyl	92	4-OHhexyl	93	5-amino-6-Brhexyl
94	4-iodo-3-Mebutyl	95	3-(neopentylamino)propyl	96	5-(Meamino)pentyl
97	2-Me-3,3-diMebutyl	98	4-Cl-3-Mepentyl	99	6-aminooctyl
100	6-amino-3-Et-4-Meoctyl	101	cycloprop-2-en-1-ylMe	102	(4-Mecyclohexyl)Me
103	Me-cyclohexyl	104	Me-cyclopentyl	105	-Bn
106	4-Me-Bn	107	4-MeO-Bn	108	4-Et-Bn
109	4-Cl-Bn	110	4-F-Bn	111	4-(F-Me)-Bn
112	3,4-diF-Bn	113	3,5-diF-Bn	114	2-Cl-4-F-Bn
115	4-Cl-2-F-Bn	116	4-Br-5-Et-2-F-Bn	117	2-F-4-Me-Bn
118	2,6-diF-Bn	119	2,4-diF-Bn	120	2,4,6-triF-Bn
121	3-(CF_3_)pyridin-2-yl	122	2-(CF_3_)pyridin-3-yl	123	3-(CF_3_)pyridin-4-yl
124	3-Br-pyridin-2-yl	125	4-MeO-3-(CF_3_)pyridin-2-yl	126	4-(CF_3_)pyridin-3-yl
127	2-(CF_3_)Ph	128	Ph	129	EtO
130	2-(6-Et-3,6-dihydro-2H-pyran-2-yl)EtO	131	4-(5-F-1H-pyrazol-1-yl)Ph	132	4-(4-F-1H-pyrazol-1-yl)Ph
133	4-(3-F-1H-pyrazol-1-yl)Ph	134	4-(3,4-diF-1H-pyrazol-1-yl)Ph	135	4-(3,4,5-triF-1H-pyrazol-1-yl)Ph
136	4-(4,5-diF-1H-pyrazol-1-yl)Ph	137	4-(3,5-diF-1H-pyrazol-1-yl)Ph	138	4-(3-Br-1H-pyrazol-1-yl)Ph
139	4-(4-Br-1H-pyrazol-1-yl)Ph	140	4-(5-Br-1H-pyrazol-1-yl)Ph	141	4-(4,5-diBr-1H-pyrazol-1-yl)Ph
142	4-(3,4-diBr-1H-pyrazol-1-yl)Ph	143	4-(3,5-diBr-1H-pyrazol-1-yl)Ph	144	4-(3,4,5-triBr-1H-pyrazol-1-yl)Ph
145	4-(5-Me-1H-pyrazol-1-yl)Ph	146	4-(4-Me-1H-pyrazol-1-yl)Ph	147	4-(3-Me-1H-pyrazol-1-yl)Ph
148	4-(3,4-diMe-1H-pyrazol-1-yl)Ph	149	4-(4,5-diMe-1H-pyrazol-1-yl)Ph	150	4-(3,5-diMe-1H-pyrazol-1-yl)Ph
151	4-(3,4,5-triMe-1H-pyrazol-1-yl)Ph	152	4-(3-iodo-1H-pyrazol-1-yl)Ph	153	4-(4-iodo-1H-pyrazol-1-yl)Ph
154	4-(5-iodo-1H-pyrazol-1-yl)Ph	155	4-(4,5-diI-1H-pyrazol-1-yl)Ph	156	4-(3,4-diI-1H-pyrazol-1-yl)Ph
157	4-(3,4,5-triiodo-1H-pyrazol-1-yl)Ph	158	4-(3,5-diI-1H-pyrazol-1-yl)Ph	159	4-(3-Cl-1H-pyrazol-1-yl)Ph
160	4-(4-Cl-1H-pyrazol-1-yl)Ph	161	4-(5-Cl-1H-pyrazol-1-yl)Ph	162	4-(4,5-diCl-1H-pyrazol-1-yl)Ph
163	4-(3,5-diCl-1H-pyrazol-1-yl)Ph	164	4-(3,4-diCl-1H-pyrazol-1-yl)Ph	165	4-(3,4,5-triCl-1H-pyrazol-1-yl)Ph
166	4-(3-amino-1H-pyrazol-1-yl)Ph	167	4-(4-amino-1H-pyrazol-1-yl)Ph	168	4-(5-amino-1H-pyrazol-1-yl)Ph
169	4-(4,5-diamino-1H-pyrazol-1-yl)Ph	170	4-(3,5-diamino-1H-pyrazol-1-yl)Ph	171	4-(3,4-diamino-1H-pyrazol-1-yl)Ph
172	4-(3,4,5-triamino-1H-pyrazol-1-yl)Ph	173	4-(3-Me-1H-pyrazol-1-yl)Ph	174	4-(4-Me-1H-pyrazol-1-yl)Ph
175	4-(5-Me-1H-pyrazol-1-yl)Ph	176	4-(4,5-diMe-1H-pyrazol-1-yl)Ph	177	4-(3,5-diMe-1H-pyrazol-1-yl)Ph
178	4-(3,4-diMe-1H-pyrazol-1-yl)Ph	179	4-(3,4,5-triMe-1H-pyrazol-1-yl)Ph	180	4-(5-Et-1H-pyrazol-1-yl)Ph
181	4-(4-Et-1H-pyrazol-1-yl)Ph	182	4-(5-Et-4-Me-1H-pyrazol-1-yl)Ph	183	4-(5-Et-3,4-diMe-1H-pyrazol-1-yl)Ph
184	4-(5-(Me-thio)-1H-pyrazol-1-yl)Ph	185	4-(4-Me-5-(Me-thio)-1H-pyrazol-1-yl)Ph	186	4-(4,5-bis(Me-thio)-1H-pyrazol-1-yl)Ph
187	4-(3-Me-4,5-bis(Me-thio)-1H-pyrazol-1-yl)Ph	188	4-(5-(aminothio)-1H-pyrazol-1-yl)Ph	189	4-(4-(aminothio)-1H-pyrazol-1-yl)Ph
190	4-(4-(aminothio)-5-ME-1H-pyrazol-1-yl)Ph	191	4-(4,5-bis(aminothio)-1H-pyrazol-1-yl)Ph	192	[1,1′-biPh]-4-yl
193	4-(5H-tetrazol-5-yl)Ph	194	4-(1H-imidazol-1-yl)Ph	195	4-(1H-1,2,4-triazol-1-yl)Ph
196	4-(1H-tetrazol-1-yl)Ph	197	4-(thiophen-2-yl)Ph	198	4-(pyridin-2-yl)Ph
199	4-(pyrazin-2-yl)Ph	200	4-(pyrimidin-2-yl)Ph	201	4-(pyridazin-3-yl)Ph
202	4-(piperazin-1-yl)Ph	203	3H-indol-2-yl	204	7H-purin-8-yl
205	1,8a-dihydroindolizin-2-yl	206	isoquinolin-6-yl	207	quinolin-6-yl
208	cyclopenta-2,4-dienecarbonyl	209	2-Mecyclopenta-2,4-dienecarbonyl	210	2-F-cyclopenta-2,4-dienecarbonyl
211	2-aminocyclopenta-2,4-dienecarbonyl	212	2-Mecyclopenta-2,4-dienecarbonyl	213	3-Mecyclopenta-2,4-dienecarbonyl
214	2,3-diMecyclopenta-2,4-dienecarbonyl	215	2-Clcyclopenta-2,4-dienecarbonyl	216	3-Clcyclopenta-2,4-dienecarbonyl
217	2,3-diClcyclopenta-2,4-dienecarbonyl	218	3-Brcyclopenta-2,4-dienecarbonyl	219	2-Brcyclopenta-2,4-dienecarbonyl
220	2,3-diBrcyclopenta-2,4-dienecarbonyl	221	2-iodocyclopenta-2,4-dienecarbonyl	222	3-iodocyclopenta-2,4-dienecarbonyl
223	2,3-diIcyclopenta-2,4-dienecarbonyl	224	amino(cyclopenta-2,4-dien-1-yl)Me	225	amino(2-F-cyclopenta-2,4-dien-1-yl)Me
226	NH_2_(2,3-diF-cyclopenta-2,4-dien-1-yl)Me	227	NH_2_(2-Mecyclopenta-2,4-dien-1-yl)Me	228	HN(2,3-diMecyclopenta-2,4-dien-1-yl)Me
229	(2,3-diMecyclopenta-2,4-dien-1-yl)(CH_3_NHMe	230	(CH_3_NH)(2-Mecyclo-penta-2,4-dien-1-yl)Me	231	(CH_3_NH)(3-Mecyclo-penta-2,4-dien-1-yl)Me
232	(3-F-cyclopenta-2,4-dien-1-yl)(CH_3_NH)Me	233	(2-F-cyclopenta-2,4-dien-1-yl)(CH_3_NH)Me	234	(2,3-diF-cyclopenta-2,4-dien-1-yl)(CH_3_NH)Me
235	(2,3-diMecyclopenta-2,4-dien-1-yl)(FNH)Me	236	FNH(2-Mecyclopenta-2,4-dien-1-yl)Me	237	FNH(3-Mecyclopenta-2,4-dien-1-yl)Me
238	F-amino(3-F-cyclopenta-2,4-dien-1-yl)Me	239	(2,3-diF-cyclopenta-2,4-dien-1-yl)(FNH)Me	240	(2,3-diClcyclopenta-2,4-dien-1-yl)(FNH)Me
241	(2-Clcyclopenta-2,4-dien-1-yl)(FNH)Me	242	(3-Clcyclopenta-2,4-dien-1-yl)(FNH)Me	243	(3-Brcyclopenta-2,4-dien-1-yl)(F-amino)Me
244	(2,3-diBrcyclopenta-2,4-dien-1-yl)(FNH)Me	245	(2-Brcyclopenta-2,4-dien-1-yl)(FNH)Me	246	NH_2_(2-carbamoylcyclopenta-2,4-dien-1-yl)Me
247	NH_2_(3-carbamoylcyclopenta-2,4-dien-1-yl)Me	248	NH_2_(2-carbamoyl-3-F-cyclopenta-2,4-dien-1-yl)Me	249	NH_2_(2-carbamoyl-3-Clcyclopenta-2,4-dien-1-yl)Me
250	NH_2_(3-NH-2-carbamoylcyclo penta-2,4-dien-1-yl)Me	251	2-carbamoylPh-HCOO^–^	252	3-carbamoylPh-HCOO^–^
253	4-carbamoylPh-HCOO^–^	254	2-MePh-HCOO^–^	255	3-MePh-HCOO^–^
256	3-MePh-HCOO^–^	257	2,3-diMePh-HCOO^–^	258	(2-carbamoylPh)(imino)Me
259	imino(Ph)Me	260	3-carbamoylPh(imino)Me	261	4-carbamoylPh(imino)Me
262	imino(2-MePh)Me	263	2,3-diMePh(imino)Me	264	imino(3-MePh)Me
265	imino(4-MePh)Me	266	(F-imino)(2-F-Ph)Me	267	F-imino(3-F-Ph)Me
268	(3-BrPh)(F-imino)Me	269	(2-BrPh)(F-imino)Me	270	(2-ClPh)(F-imino)Me
271	(3-ClPh)(F-imino)Me	272	Brimino(3-ClPh)Me	273	Brimino(3-BrPh)Me
274	Cl-imino(3-ClPh)Me	275	Cl-imino(2-ClPh)Me	276	imino(o-tolyl)Me
277	imino(2-(CF_3_)Ph)Me	278	imino(3-(CF_3_)Ph)Me	279	3-formylbenzamide
280	2-formylbenzamide	281	4-formylbenzamide	282	-Bz-2-Me
283	-Bz-2,3-diMe	284	-Bz-3-Me	285	-Bz-4-Me
286	-Bz-2-Me	287	-Bz-2-(CF_3_)	288	-Bz-3-(CF_3_)
289	-Bz-2-F-	290	NH_2_(3-Br-2-carbamoylcyclopenta-2,4-dien-1-yl)Me	291	Carbamoyl
292	4-Cl-1H-pyrazol-1-yl	293	4,5-diCl-1H-pyrazol-1-yl	294	5-Cl-1H-pyrazol-1-yl
295	3-Cl-1H-pyrazol-1-yl	296	3-Br-1H-pyrazol-1-yl	297	4-Br-1H-pyrazol-1-yl
298	5-Br-1H-pyrazol-1-yl	299	4,5-diBr-1H-pyrazol-1-yl	300	3,4,5-triBr-1H-pyrazol-1-yl
301	4-Me-1H-pyrazol-1-yl	302	4,5-diMe-1H-pyrazol-1-yl	303	5-Me-1H-pyrazol-1-yl
304	5-iodo-1H-pyrazol-1-yl	305	4-iodo-1H-pyrazol-1-yl	306	3-iodo-1H-pyrazol-1-yl
307	3,4-diI-1H-pyrazol-1-yl	308	3,4,5-triiodo-1H-pyrazol-1-yl	309	3,4,5-triF-1H-pyrazol-1-yl
310	3-F-1H-pyrazol-1-yl	311	3,4-diF-1H-pyrazol-1-yl	312	4-F-1H-pyrazol-1-yl
313	5-F-1H-pyrazol-1-yl	314	3-NH_2_-1H-pyrazol-1-yl	315	4-amino-1H-pyrazol-1-yl
316	5-amino-1H-pyrazol-1-yl	317	5-Me-1H-pyrazol-1-yl	318	5-Et-1H-pyrazol-1-yl
319	4-Me-1H-pyrazol-1-yl	320	4,5-diMe-1H-pyrazol-1-yl	321	5-(Me-Me)-1H-pyrazol-1-yl
322	4-Me-5-(Me-Me)-1H-pyrazol-1-yl	323	5-(H_2_N-thio)-4-Me-1H-pyrazol-1-yl	324	4,5-bis(aminothio)-1H-pyrazol-1-yl
325	4,5-bis(H_2_N-thio)-3-Me-1H-pyrazol-1-yl	326	5-Et-4-Me-1H-pyrazol-1-yl	327	pyridazin-3-yl
328	pyridazin-4-yl	329	pyrimidin-4-yl	330	1,3,5-triazin-2-yl
331	pyrimidin-2-yl	332	pyrazin-2-yl	333	Cyclohexyl
334	piperidin-1-yl	335	tetrahydropyridazin-1(2H)-yl	336	piperazin-1-yl
337	1,2,4-triazinan-1-yl	338	2-(thiazol-2-yl)butyl	339	2-(thiazol-2-yl)pentyl

^a^ All fragments were used for substitutions in the R_1_ and R_2_ positions.

**Table 6:**
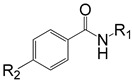
GFE and their components for the top scoring 90 virtual BHMB analogs. The analog numbering concatenates the index of each substituent R_1_ to R_2_ with the substituent numbers taken from Table 5 except for hydrogen which is directly specified by the letter H.

Designed Analogs		M_w_^a^ [g⋅mol^−1^]	ΔΔH_MM_^b^ [kcal⋅mol^−1^]	ΔΔG_sol_^c^ [kcal⋅mol^−1^]	ΔΔTS_vib_^d^ [kcal⋅mol^−1^]	ΔΔG_com_^e^ [kcal⋅mol^−1^]	IC_50_^pre f^ [nM]
N	BHMB1	385	0	0	0	0	20 ^g^
1	32–218	389	−21.96	14.78	0.15	−7.33	0.24
2	39–95	359	21.7	2.26	1.98	21.99	1996
3	40–232	439	−1.72	5.01	−1.65	4.94	200
4	40–234	405	−10.02	2.93	0.99	−8.08	0.16
5	41–93	409	−11.28	3.31	2.16	−10.14	0.052
6	42–207	342	−7.22	6.24	−4.45	3.47	90
7	47–235	421	−5.66	3.16	−5.73	3.24	80
8	54–282	505	−3.55	6.63	−3.02	6.1	370
9	65–235	413	−5.71	3.01	1.99	−4.69	1
10	67–67	375	−8.72	3.21	0.32	−5.83	0.55
11	67–212	505	0.56	2.82	−2.2	5.59	280
12	67–215	599	3.45	3.22	−2.52	9.18	1990
13	70–241	430	−13.98	14.15	−1.02	1.18	30
14	72–232	401	−10.09	4.74	−0.7	−4.65	1
15	79–9	402	−12.2	15.92	−2.82	6.54	470
16	79–92	439	−13.99	6.17	3.15	−10.97	0.033
17	79–215	683	−4.32	2.82	−5.06	3.56	94
18	79–338	375	−12.46	5.35	3.19	−10.3	0.047
19	79–339	389	−13.64	5	2.01	−10.65	0.039
20	86–233	393	−14.09	17.65	−2.46	6.02	350
21	87–24	410	−8.64	4.95	1.59	−5.28	0.74
22	87–122	480	−9.46	4.77	0.39	−5.07	0.83
23	87–205	450	−13.78	17.73	−4.81	8.75	1570
24	87–208	453	−7.19	3.56	−4.64	1.01	20
25	92–169	406	−14.2	14.73	5.02	−4.48	2
26	95–215	606	−6.76	1.94	−2.28	−2.53	4
27	106–215	605	−6.81	2.61	−6.96	2.76	60
28	106–233	407	−7.12	5.75	−3.77	2.4	50
29	106–241	432	−2.77	6.09	−2.2	5.51	270
30	111–95	389	9.21	−0.51	0.18	8.52	1380
31	112–208	388	2.68	0.15	−2.26	5.09	210
32	114–232	460	−11.36	15.18	−4.36	8.18	1150
33	118–84	443	−6.36	4.46	−0.67	−1.23	7
34	118–235	500	−5.5	1.49	−3.13	−0.89	9
35	122–57	400	−12.27	15.46	1.16	2.03	40
36	122–88	445	−11.79	16.54	−0.93	5.68	290
37	122–92	390	−18.45	16.69	7.09	−8.85	0.1
38	122–282	504	−6.19	12.29	2.79	3.31	81
39	128–232	493	−19.75	17.06	−6.07	3.38	83
40	138–65	416	−18.69	16.51	−0.24	−1.94	5
41	147–282	744	−16.78	13.56	−7.11	3.89	110
42	158–209	453	−20.41	19.64	−2.82	2.05	41
43	166–92	393	−14.08	7.1	1.14	−8.12	0.15
44	169–232	485	−18.91	17.42	−5.38	3.9	113
45	172–235	495	−23.56	16.58	−4.17	−2.81	3
46	174–214	537	−15.39	17.46	−0.99	3.06	71
47	174–311	399	−20.87	18.65	−2.04	−0.18	12
48	177–282	571	−15.7	15.55	−7.49	7.34	725
49	178–233	515	−22.75	17.89	−5.82	0.96	23
50	185–282	494	−10.48	15.12	−4.02	8.66	1514
51	201–15	315	−15.79	15.32	3.25	−3.72	2
52	214–233	499	3.45	2.69	−2.89	9.03	1820
53	220–99	420	−7.16	3.71	5.22	−8.66	0.11
54	225–233	424	−7.4	15.23	−1.23	9.05	1863
55	232–276	451	−19	17.57	−0.81	−0.63	2
56	234–99	408	−8.67	3.96	4.31	−9.02	0.09
57	234–234	426	−5.74	17.27	2.21	9.32	2138
58	238–25	398	−5.51	5.21	8.81	−9.11	0.09
59	240–84	423	−15.36	16.82	−0.29	1.74	34
60	241–63	396	−7.77	19.23	5.04	6.42	437
61	244–99	426	−9.46	6.19	0.9	−4.17	2
62	245–43	394	−14.47	19.17	−0.26	4.96	200
63	245–174	483	−19.31	16.99	−4.81	2.48	52
64	261–212	585	−4.95	4.87	−8.29	8.21	1175
65	266–210	478	−3.42	4.54	−5.75	6.88	563
66	269–215	650	−4.2	3.14	−5.61	4.54	159
67	269–233	452	−12.9	5.61	−3.64	−3.65	2
68	269–236	575	−6.69	4.6	−5.62	3.53	92
69	270–232	486	−8.35	5.48	−6.4	3.53	92
70	279–214	525	−5.1	−0.63	−4.71	−1.02	8
71	279–282	522	−2.8	5.06	−4.06	6.32	417
72	282–26	448	−13.63	18.31	5.63	−0.95	8
73	282–280	522	−10.39	2.59	−3.51	−4.3	2
74	297–28	423	−5.81	3.84	2.05	−4.01	2
75	307–99	343	−7.43	6.73	3.3	−4.01	2
76	308–25	342	−6.7	6.12	3.74	−4.32	2
77	310–99	357	−10.86	4.79	2.67	−8.74	0.11
78	310–74	525	3.9	1.65	−3.08	8.63	1479
79	310–209	390	2.99	−0.1	−3.93	6.82	550
80	310–289	374	2.6	−0.5	−1.85	4.1	124
81	313–278	365	−20.4	18.73	−0.79	−0.88	9
82	316–150	689	−17.61	14.38	−8.08	4.85	187
83	317–98	446	−11.26	17.19	1.24	4.69	170
84	318–79	447	−10.86	9.09	−1.71	−0.07	13
85	318–89	365	−4.96	3.62	1.15	−2.48	4
86	318–198	370	0.06	4.96	1.85	3.17	75
87	318–207	370	−11.32	5.52	1.37	−7.17	0.26
88	318–212	493	−9.55	3.93	−1.59	−4.02	2
89	320–19	376	3.36	3.01	4.03	2.34	48
90	321–25	339	−1.01	2.16	4.2	−3.05	3

^a^ M_w_ is molar mass of inhibitor; ^b^ ΔΔH_MM_ is the relative enthalpic contribution to the GFE change of the InhA-BHMB complex formation ΔΔG_com_ (for details see footnote pf Table 2); ^c^ ΔΔG_sol_ is the relative solvation GFE contribution to ΔΔG_com_; ^d^ ΔΔTS_vib_ is the relative (vibrational) entropic contribution to ΔΔG_com_; ^e^ ΔΔG_com_ is the relative Gibbs free energy change related to the enzyme–inhibitor InhA-BHMB complex formation ΔΔG_com_ ≅ ΔΔH_MM_ + ΔΔG_sol_ − ΔΔTS_vib_; ^f^ IC_50_^pre^ is the predicted inhibition potency towards *Mt*InhA calculated from ΔΔG_com_ using correlation Equation B, Table 3; ^g^ IC_50_^exp^ [19] is given for the reference inhibitor BHMB1 instead of the IC_50_^pre^.

**Table 7:** ADME-related properties of the best designed BHMB analogs and known antituberculotic agents either in clinical use or currently undergoing clinical testing computed by QikProp [33].

BHMBx ^a^	#stars ^b^	M_w_^c^ [g.mol^−1^]	S_mol_^d^ [Å^2^]	S_mol,hfo_^e^ [Å^2^]	V_mol_^f^ [Å^3^]	RotB ^g^	HB_don_^h^	HB_acc_^i^	logP_o/w_^j^	logS_wat_^k^	logK_HSA_^l^	logB/B ^m^	BIP_caco_^n^ [nm.s^−1^]	#meta ^o^	IC_50_^pre^ [nM]	HOA^q^	%HOA^r^
32–218	0	389	760	380.1	1340	10	3	3.5	5.1	−5.8	0.94	−0.49	320.6	4	0.24	3	89
40–234	1	405	703	285.9	1253	6	2	3.5	5.8	−7.2	1	−0.11	2551.9	8	0.16	1	100
41–93	0	409	691	230.2	1218	11	3	3.5	4.5	−4.6	0.57	−0.42	301.4	5	0.05	3	100
67–67	1	375	716	305.9	1278	8	1	4	5.8	−6.5	0.98	−0.42	3493.1	4	0.55	1	100
79–92	0	439	737	323.6	1306	12	2	5.7	4.8	−5.9	0.48	−1.01	1105.1	4	0.033	3	100
79–338	0	375	680	367.1	1268	12	2	5.7	4.5	−4.7	0.41	−0.83	1688.3	4	0.047	3	100
79–339	0	389	760	431.0	1368	13	2	5.7	5	−5.9	0.6	−1.15	1371.3	4	0.039	3	100
87–24	0	410	701	332.5	1226	9	1	4.5	4.8	−6.3	0.68	−0.96	882.4	3	0.74	1	100
87–122	0	480	762	347.2	1363	10	1	6.95	5	−6.3	0.5	−0.62	1884.2	6	0.83	1	100
122–92	0	390	808	522.4	1430	13	2	7.6	4.5	−5.9	0.39	−1.28	1347.2	6	0.10	3	100
166–92	0	393	739	244.7	1324	10	4	5.7	3.9	−5.5	0.44	−1.83	254.9	3	0.15	3	92
234–99	0	408	730	318.4	1343	13	4	4.5	4.5	−4.4	0.56	−0.79	180.9	5	0.09	3	93
310–74	1	525	797	239.5	1419	8	2	5	6	−7.8	1.07	−0.81	1168.4	3	0.11	1	91
318–207	0	370	683	215.9	1198	6	1	5.5	4.3	−6	0.53	−0.73	1021.2	3	0.26	3	100
Rifampin	1	137.1	314	0.0	480 *	2	3	4.5	−0.7	0	−0.8	−0.8	267.5	2	−	2	67
Isoniazid	4	123.1 *	300	0.0	443 *	1	2	5	−0.6	−0.5	−0.8	−0.7	298.4	4	−	2	67
Ethambutol	2	204.3	476	395.8	806	11	4	6.4	−0.2	0.6	−0.8	0.0	107.8	4	−	2	62
Pyrazinamide	10	823.0 *	1090 *	850.0 *	2300 *	25 *	6	20.3 *	3.0	−3.1	−0.3	−2.7	38.2	11^ *^	−	1	34
Gatifloxacin	0	375.4	598	355.7	1093	2	1	6.8	0.5	−4.0	0	−0.6	17.0	1	−	2	52
Moxifloxacin	0	401.4	642	395.6	1168	2	1	6.8	1.0	−4.7	0.2	−0.6	20.9	1	−	2	56
Rifapentine	10	877.0 *	1025 *	844.9 *	2333 *	24 *	6	20.9 *	3.6	−2.2	−0.2	−1.5	224.0	13^ *^	−	1	51
Bedaquiline	4	555.5	787	213.7	1532	9	1	3.8	7.6 *	−6.9	1.7	0.4	1562.2	5	−	1	100
Delamanid	2	534.5	796	284.4	1470	7	0	6.0	5.8	−7.6	1.0	−1.0	590.9	2	−	1	85
Linezolid	0	337.4	555	337.2	996	2	1	8.7	0.6	−2.0	−0.7	−0.5	507.0	2	−	3	79
Sutezolid	1	353.4	594	330.6	1047	2	1	7.5	1.3	−3.4	−0.4	−0.4	449.3	0	−	3	82
Ofloxacin	1	361.4	581	337.0	1044	1	0	7.3	−0.4	−2.8	−0.5	−0.4	25.9	1	−	2	50
Amikacin	14	585.6	739	350.3	1500	22^ *^	17 *	26.9 *	−7.9^ *^	−0.2	−2.1	−3.5	0	14^ *^	−	1	0
Kanamycin	10	484.5	656	258.9	1291	17^ *^	15 *	22.7 *	−6.7 *	2.0	−1.4	−3.1	0	12^ *^	−	1	0
Imipenem	0	299.3	487	259.1	880	8	3	7.2	1.0	−1.8	−0.7	−1.4	35.0	3	−	3	61
Amoxicillin	2	365.4	561	164.6	1033	6	4.25	8.0	−2.5	−0.8	−1.1	−1.5	1.0	5	−	1	12
Clavulanate	0	199.2	397	184.6	630	4	2	6.5	−0.8	0.3	−1.3	−1.3	13.3	2	−	2	42

^a^ designed BHMB analogs and known antituberculotic agents, Table 6; ^b^ drug likeness, number of property descriptors (24 out of the full list of 49 descriptors of QikProp, ver. 3.7, release 14) that fall outside of the range of values for 95% of known drugs; ^c^ molar mass in [g.mol-1] (range for 95% of drugs: 130–725 g.mol^−1^) [33]; ^d^ total solvent-accessible molecular surface, in [Å2] (probe radius 1.4 Å) (range for 95% of drugs: 300–1000 Å2); ^e^ hydrophobic portion of the solvent-accessible molecular surface, in [Å2] (probe radius 1.4 Å) (range for 95% of drugs: 0–750 Å2); ^f^ total volume of molecule enclosed by solvent-accessible molecular surface, in [Å3] (probe radius 1.4 Å) (range for 95% of drugs: 500–2000 Å3); ^g^ number of non-trivial (not CX3), non-hindered (not alkene, amide, small ring) rotatable bonds (range for 95% of drugs: 0–15); ^h^ estimated number of hydrogen bonds that would be donated by the solute to water molecules in an aqueous solution. Values are averages taken over a number of configurations, so they can assume non-integer values (range for 95% of drugs: 0.0–6.0); ^i^ estimated number of hydrogen bonds that would be accepted by the solute from water molecules in an aqueous solution. Values are averages taken over a number of configurations, so they can assume non-integer values (range for 95% of drugs: 2.0–20.0); ^j^ logarithm of partitioning coefficient between n-octanol and water phases (range for 95% of drugs: −2 to 6.5); ^k^ logarithm of predicted aqueous solubility, logS. S in [mol·dm–3] is the concentration of the solute in a saturated solution that is in equilibrium with the crystalline solid (range for 95% of drugs: −6.0 to 0.5); ^l^ logarithm of predicted binding constant to human serum albumin (range for 95% of drugs: −1.5 to 1.5); ^m^ logarithm of predicted brain/blood partition coefficient (range for 95% of drugs: −3.0 to 1.2); ^n^ predicted apparent Caco-2 cell membrane permeability in Boehringer-Ingelheim scale in [nm s-1] (range for 95% of drugs: < 25 poor, > 500 nm s^−1^ great); ^o^ number of likely metabolic reactions (range for 95% of drugs: 1–8); ^p^ predicted inhibition constants IC50pre. IC50pre was predicted from computed ΔΔGcom using the regression Equation B shown in Table 3; ^q^ human oral absorption (1 = low, 2 = medium, 3 = high); r percentage of human oral absorption in gastrointestinal tract (<25% = poor, >80% = high); * star in any column indicates that the property descriptor value of the compound falls outside the range of values for 95% of known drugs.

**Table 8:** GFE, its components, and predicted InhA inhibitory potencies of 24 approved drugs which contain benzamide scaffold in their molecular structure (for 2D representation see Table 9).

DrugBank Accession Number	Name ^a^	M_w_ [g⋅mol^−1^]	ΔΔH_MM_ [kcal⋅mol^−1^]	ΔΔG_sol_ [kcal⋅mol^−1^]	ΔΔTS_vib_ [kcal⋅mol^−1^]	ΔΔG_com_ [kcal⋅mol^−1^]	IC_50_^pre^ [nM]
DB00345	Aminohippuric Acid	194	10.6	3.81	2.03	12.39	11,370
DB00391	Sulpiride	341	4.53	3.59	3.91	4.2	132
DB00409	Remoxipride	371	11.49	−1.65	7.06	2.78	60
DB00604	Cisapride	465	8.47	−0.06	2.97	5.44	258
DB00619	Imatinib	493	−3.54	13.79	2.56	7.69	879
DB01035	Procainamide	235	12.97	2.22	6.14	9.05	1 847
DB01168	Procarbazine	221	16.54	1.73	2.12	16.15	88,971
DB01171	Moclobemide	268	−7.79	16.78	3.22	5.76	308
DB01233	Metoclopramide	299	−4.54	17.82	6.47	6.81	544
DB01393	Bezafibrate	361	−11.95	18.14	1.32	4.87	189
DB06288	Amisulpride	369	4.91	5.02	6.5	3.42	86
DB06421	Declopramide	269	−6.29	17.29	1.34	9.66	2577
DB06422	Tricalopride	313	−13.62	17.61	4.43	−0.44	10
DB06626	Axtinib	386	5.04	0.12	−0.46	5.62	281.8
DB07069	3-Hydroxyhippuric Acid	195	0.99	5.78	1.41	5.36	247
DB08950	Indoramin	347	−2.33	18.92	4.19	12.39	11,495
DB09018	Bromopride	344	−8.55	17.12	3.08	5.49	265
DB11282	Diethyltoluamide	191	2.37	2.47	5.48	−0.64	9
DB11480	Zoalene	225	−0.72	7.5	0	6.78	535
DB12518	Raclopride	347	−12	18.54	1.78	4.77	178
DB13025	Tiapride	328	5.98	2.72	4.83	3.86	109
DB13273	Sultopride	354	−17.19	19.7	6.23	−3.42	1.7
DB13523	Veralipride	384	0.93	4.96	4.84	1.05	23
*DB15445*	Iodohippuric Acid	305	1.09	6.93	−1.76	9.78	2751

^a^ for definition of the individual quantities see the footnote of Table 2.

**Table 9:** Representation for 24 approved drugs containing benzamide scaffold listed in Table 8.

**DB00345**	**DB00391**	**DB00409**	**DB00604**	**DB00619**	**DB01035**	**DB01168**	**DB01171**
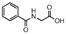	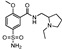	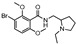	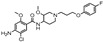	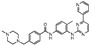	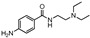	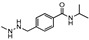	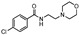
**DB01233**	**DB01393**	**DB06288**	**DB06421**	**DB06422**	**DB06626**	**DB07069**	**DB08950**
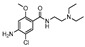	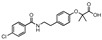	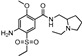	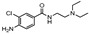	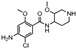	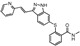	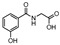	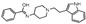
**DB09018**	**DB11282**	**DB11480**	**DB12518**	**DB13025**	**DB13273**	**DB13523**	**DB15445**
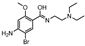	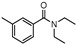	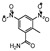	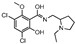	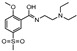	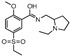	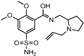	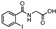

## Abbreviations

2D	Two-dimensional
3D	Three-dimensional
ADME	Absorption, distribution, metabolism, and excretion
AHO	Hydroxamic acids inhibitors
BHMBx	Training set of *N*-benzyl-4-((heteroaryl)methyl)benzamides
BHMVx	Validation set of *N*-benzyl-4-((heteroaryl)methyl)benzamides
CAMD	computer-aided molecular design
E_int_	MM enzyme–inhibitor interaction energy per residue
ΔΔG_com_	Relative complexation GFE
GFE	Gibbs free energy
ΔΔG_sol_	Relative solvation GFE
HBA	Hydrogen bond Acceptor
HBD	Hydrogen bond Donor
H_MM_	Enthalpy component of GFE
HOA	Human oral absorption
HYD	Hydrophobic
HYDA	Hydrophobic Aliphatic
IC_50_	Half-maximal inhibitory concentration
IE	Interaction energy
InhA	2-trans enoyl-acyl carrier protein reductase
KatG	*Mycobacterium tuberculosis* catalase–peroxidase
LHP	Large hydrophobic pocket
LOO	Leave-one-out cross-validation
MM	Molecular mechanics
MM-PB	Molecular mechanics–Poisson–Boltzmann
*Mt*	*Mycobacterium tuberculosis*
*Mt*InhA	2-trans enoyl-acyl carrier protein reductase of *Mycobacterium tuberculosis*
PDB	Protein Data Bank
*Pf*EACP	2-trans enoyl-acyl carrier protein reductase of *Plasmodium falciparum*
PH4	Pharmacophore
QSAR	Quantitative structure–activity relationships
RMSD	Root-mean square deviation
SAR	Structure–activity relationships
TB	Tuberculosis
TS	Training set
VS	Validation set
